# Arsenic Trioxide Suppressed Migration and Angiogenesis by Targeting FOXO3a in Gastric Cancer Cells

**DOI:** 10.3390/ijms19123739

**Published:** 2018-11-24

**Authors:** Lin Zhang, Lei Liu, Shining Zhan, Lili Chen, Yueyuan Wang, Yujie Zhang, Jun Du, Yongping Wu, Luo Gu

**Affiliations:** 1 Department of Biochemistry and Molecular Biology, Nanjing Medical University, Nanjing 211166, China; zhanglinyantai@126.com (L.Z.); liul0131@126.com (L.L.); 2 Department of Pathology, Xuzhou Medical University, Xuzhou 221004, China; zhanshining@163.com (S.Z.); lilychen1210@126.com (L.C.); wyp@xzhmu.edu.cn (Y.W.); 3 Department of Physiology, Nanjing Medical University, Nanjing211166, China; yueyuanxljm@126.com (Y.W.); zeater87@126.com (Y.Z.); dujun@njmu.edu.cn (J.D.); 4 Jiangsu Key Lab of Cancer Biomarkers, Prevention and Treatment, Collaborative Innovation Center for Cancer Personalized Medicine, Nanjing Medical University, Nanjing 211166, China

**Keywords:** As_2_O_3_, FOXO3a, gastric cancer, migration, angiogenesis

## Abstract

Arsenic trioxide (As_2_O_3_), a traditional remedy in Chinese medicine, has been used in acute promyelocytic leukemia (APL) research and clinical treatment. Previous studies have shown that As_2_O_3_ exerts its potent antitumor effects in solid tumors by regulating cell proliferation and survival. The aim of this study was to investigate whether As_2_O_3_ inhibited gastric cancer cell migration and angiogenesis by regulating FOXO3a expression. We found that As_2_O_3_ reduced gastric cancer cell viability in a dose-dependent manner and also inhibited cell migration and angiogenesis in vitro. Western blotting and immunofluorescence showed that As_2_O_3_ downregulated the levels of p-AKT, upregulated FOXO3a expression in the nucleus, and attenuated downstream Vascular endothelial growth factor (VEGF) and Matrix metallopeptidase 9 (MMP9) expression. Moreover, we demonstrated that knockdown of FOXO3a significantly reversed the inhibition of As_2_O_3_ and promoted cell migration and angiogenesis in vitro. Further, As_2_O_3_ significantly inhibited xenograft tumor growth and angiogenesis by upregulating FOXO3a expression in vivo. However, knockdown of FOXO3a attenuated the inhibitory effect of As_2_O_3_ in xenograft tumors, and increased microvessel density (MVD) and VEGF expression. Our results demonstrated that As_2_O_3_ inhibited migration and angiogenesis of gastric cancer cells by enhancing FOXO3a expression.

## 1. Introduction

Arsenic trioxide (As_2_O_3_) is a naturally occurring arsenic compound and has been used as a drug in traditional Chinese medicine for thousands of years [1]. As_2_O_3_ was able to induce complete remission in patients with APL without severe toxicity, so it has been clinically used to treat APL [2]. At present, As_2_O_3_ has been documented to exhibit a tumor-suppressive function in several human cancers, such as hepatocellular carcinoma, breast cancer, ovarian cancer, lung cancer, and so on [3,4,5]. A low dosage of As_2_O_3_ inhibited angiogenesis in epithelial ovarian cancer without cell viability or apoptosis [6]. As_2_O_3_ inhibited cell viability and induced apoptosis by reactivating the Wnt inhibitor secreted frizzled-related protein-1 in human prostate cancer cells [7]. In the present study, we tried to investigate the effect of As_2_O_3_ on the migration and angiogenesis of gastric cancer cells and its underlying molecular mechanism.

FOXO3a, a member of the forkhead box O transcription factor family, is well known to regulate numerous genes through its transcriptional activity. FOXO3a was shown to be involved in the regulation of cell cycle arrest, longevity, oxidative stress, DNA damage, and immune dysfunction [8,9]. Recent studies suggested that FOXO3a behaves as a tumor suppressor in various cancers and plays key roles in cancer progression and metastasis [10,11]. FOXO3a overexpression was correlated with gefitinib sensitivity, the suppression of cancer stemness, and better progression-free survival in lung cancer patients [12]. High nuclear FOXO3a expression was inversely correlated with prostate cancer prognostic markers such as high preoperative prostatic specific antigen (PSA) and a higher Gleason grade [13]. In contrast, FOXO3a promoted glioma cell proliferation and invasion through regulation of cell arrest and induction of MMP9 expression [14]. FOXO3a overexpression was also associated with glioblastoma progression and independently indicated poor prognosis in patients [15]. Our laboratory previous study showed FOXO3a regulated cell growth arrest and sensitivity of breast cancer cells treated with As_2_O_3_ [16]. These studies suggest that FOXO3a might play variable but important functional roles in tumor progression and chemotherapy.

Tumor angiogenesis is an essential process involving the formation of new blood vessels and plays a critical role in tumor growth and progression [17]. Vascular endothelial growth factor (VEGF), also known as VEGF-A, is a key regulator of angiogenesis in physiological and pathological processes [18]. VEGF stimulates the proliferation and migration of endothelial cells and recruits endothelial progenitor cells into tumors for vascular formation. VEGF also directly affects tumor cells and is associated with the initiation, progression, and recurrence of tumors [19,20]. Blocking VEGF to inhibit tumor angiogenesis has been used an attractive therapeutic approach in treating glioblastoma and lung cancer [21,22]. In the present study, we investigated the effects of As_2_O_3_ on the migration and angiogenesis of gastric cancer cells. 

## 2. Results

### 2.1. As_2_O_3_ Inhibited Gastric Cancer Cell Viability in a Dose-Dependent Manner

**Figure 1:**
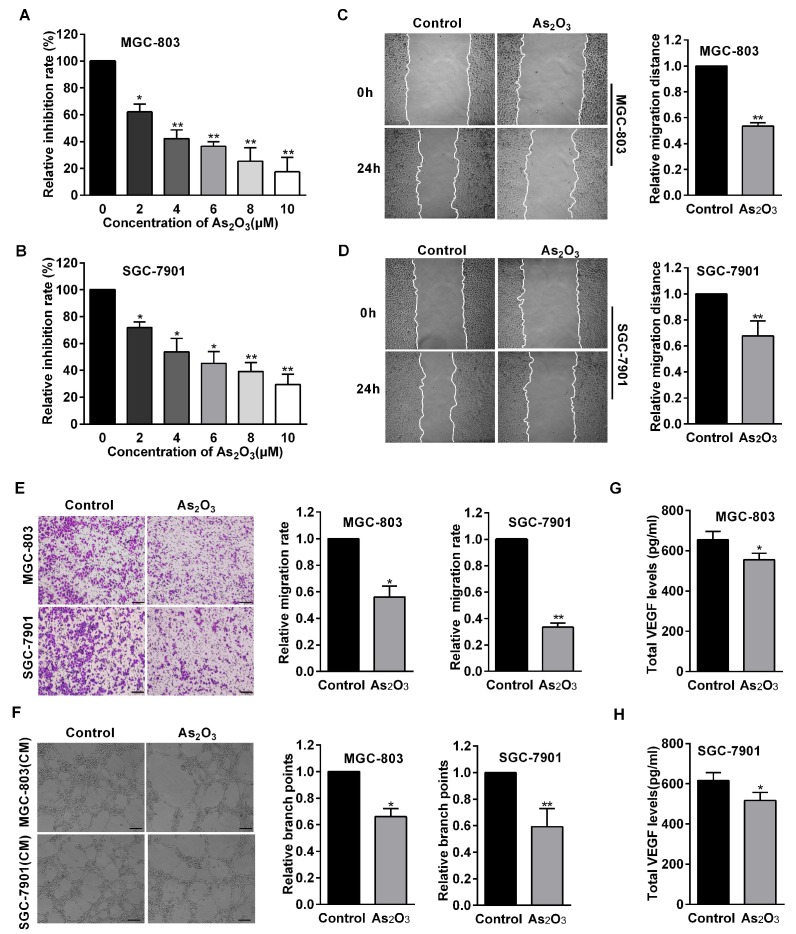
The effects of As_2_O_3_ on gastric cancer cell viability, migration, and angiogenesis. (**A**,**B**) Cell Counting Kit-8 assays were used to measure cell viability in MGC-803 and SGC-7901 cells treated with different concentrations of As_2_O_3_ for 24 h. (**C**,**D**) Wound healing assays were used to detect migration distances of these cells treated with As_2_O_3_ for 24 h (100×). The quantification of migration rates was analyzed, respectively. (**E**) Transwell assay were performed to measure cell migration in gastric cancer cells treated with As_2_O_3_. The quantification of migration cells was performed. Scale bar, 100 µm. (**F**) Tube formation assays detected formed tubes of Human umbilical vein endothelial cells (HUVECs) treated with conditioned medium. Calculating branch points per field was used to quantify the ability of tube formation. Scale bar, 100 µm. (**G**,**H**) ELISA analysis detected VEGF protein secretion. All data were obtained from three independent experiments. Compared with the control group, * *p* < 0.05, ** *p* < 0.01.

To examine whether As_2_O_3_ could suppress gastric cancer cell viability, Cell Counting Kit-8 (CCK8) assays were used to measure cell growth inhibition in MGC-803 and SGC-7901 cells treated with different concentrations of As_2_O_3_. The results showed that As_2_O_3_ significantly suppressed cell viability in a dose-dependent manner compared with the control group (Figure 1A,B). Specifically, we found that 4 μM of As_2_O_3_ was the lowest concentration that could make the cell inhibition rate reach about 50%. Moreover, more than 8 μM of As_2_O_3_ had a significant toxic effect on cell death. Therefore, we chose 4 μM of As_2_O_3_ as the optimal concentration in this experiment. 

### 2.2. As_2_O_3_ Inhibited Cell Migration and Endothelial Cell Tube Formation In Vitro

Wound healing assays were used to measure cell motility, and the results demonstrated that As_2_O_3_ inhibited cell motility in MGC-803 and SGC-7901 cells. (Figure 1C,D). Transwell assays were used to detect whether As_2_O_3_ inhibited cell migration activity. We observed that the numbers of migratory cells significantly decreased after As_2_O_3_ treatment (Figure 1E). The results indicated that As_2_O_3_ played a negative role in regulating gastric cancer cell migratory potential. Angiogenesis was considered to be crucial for metastasis and progression of cancer and was involved in the carcinogenesis of gastric cancer. We then examined whether As_2_O_3_ could affect angiogenesis using an in vitro human umbilical vein endothelial cells (HUVECs) model. The results showed As_2_O_3_ decreased gastric cancer cells to induce tube formation of HUVECs, suggesting that As_2_O_3_ inhibited gastric cancer angiogenesis in vitro (Figure 1F). In addition, enzyme-linked immunoabsorbent assay (ELISA) indicated As_2_O_3_ significantly decreased VEGF secretion levels in MGC-803 and SGC-7901 cells compared with the control group (Figure 1G,H). These results indicated that As_2_O_3_ inhibited gastric cancer cell migration and angiogenesis in vitro. 

### 2.3. The Antitumor Effect of As_2_O_3_ Was Mediated by FOXO3a

**Figure 2:**
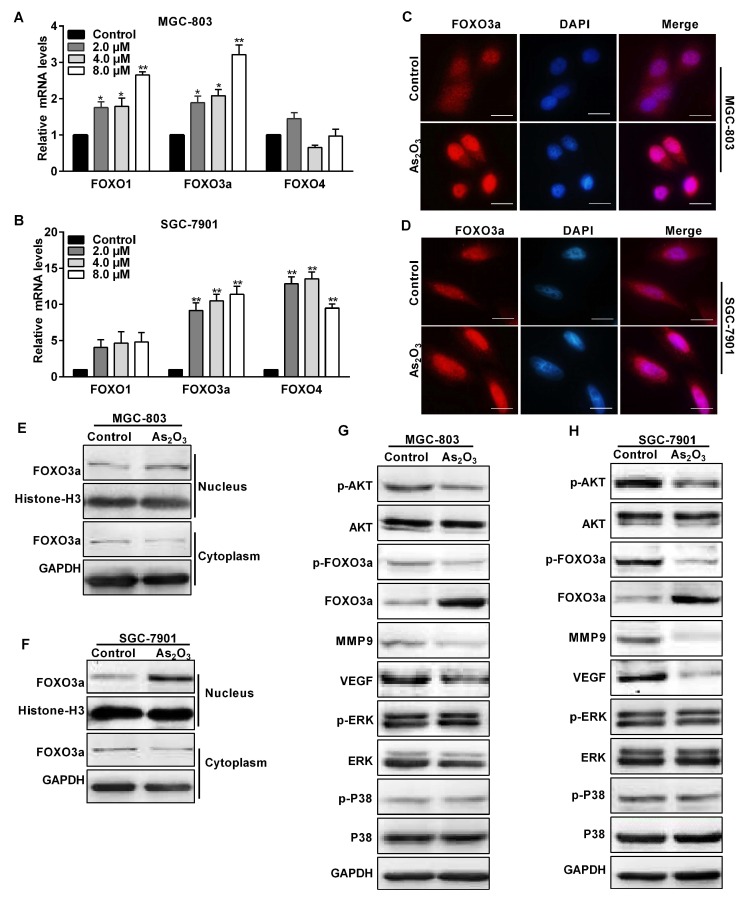
As_2_O_3_ distinctly upregulated the expression of FOXO3a in the nucleus to regulate signal associated proteins. (**A**,**B**) qRT-PCR analyses showed FOXO transcription factor family mRNA expression levels. Average FOXO1, FOXO3a, and FOXO4 mRNA levels were normalized to GAPDH. (**C**,**D**) Representative immunofluorescence images showed FOXO3a expression was mainly in the nucleus (red light). Cell nuclei were labelled with DAPI. Scale bar, 5 μm. (**E**,**F**) Western blotting analysis showed the expression of FOXO3a in MGC-803 and SGC-7901 cells treated with As_2_O_3_. FOXO3a in the cytoplasm and nucleus were extracted, respectively. (**G**,**H**) Protein levels of p-AKT, AKT, p-FOXO3a, FOXO3a, VEGF, MMP9, p-ERK, ERK, p-P38, and P38 were detected by western blotting analysis. The whole cell lysate protein was extracted from these cells treated with As_2_O_3_ for 24 h. Compared with the control group, * *p* < 0.05, ** *p* < 0.01.

It is known that FOXO3a plays an antitumor role in human cancers. Thus, we wondered whether FOXO3a mediated As_2_O_3_ antitumor activity in gastric cancer cells. We measured the forkhead box O transcription factor family mRNA levels in MGC-803 and SGC-7901 cells treated with different concentrations of As_2_O_3_. The mRNA levels FOXO1, FOXO3a, and FOXO4 were differently increased in these cells treated with As_2_O_3_. The levels of FOXO1and FOXO4 were slightly increased, and there was not statistically significant difference compared with the control group. The increased levels of FOXO3a were the most significant. Moreover, the FOXO3a mRNA level distinctly elevated in gastric cancer cells treated with 4 μM of As_2_O_3_ (Figure 2A,B). This was consistent with the above experimental results. Immunofluorescence staining showed that FOXO3a was mainly located in the nucleus, and the average fluorescence density of FOXO3a was remarkably higher in these cells treated with As_2_O_3_ (Figure 2C,D). Then, we respectively extracted nuclear and cytosolic protein. The results showed As_2_O_3_ distinctly upregulated FOXO3a expression in the nucleus, while it downregulated FOXO3a expression in the cytoplasm (Figure 2E,F). The FOXO3a located in the nucleus was a functional form that had the function of inhibiting tumors. As_2_O_3_ increased FOXO3a expression in the nucleus and played a role of tumor inhibition. Then, we continued to study the mechanism of As_2_O_3_ in gastric cancer cells. AKT is one of the most important regulators of FOXO3a. Western blotting detected p-AKT/AKT, p-ERK/ERK, and p-P38/P38 signaling pathways and migration and angiogenesis related MMP9 and VEGF expression. We found that As_2_O_3_ regulated FOXO3a phosphorylation by attenuating p-AKT expression, but it had no obvious effect on the p-ERK/ERK and p-P38/P38 signaling pathways (Figure 2G,H). Therefore, the results showed that As_2_O_3_ regulation FOXO3a expression depended on the AKT pathway, and then decreased MMP9 and VEGF expression to inhibit cell migration and angiogenesis. 

### 2.4. FOXO3a Participated in the Inhibitory Effect of As_2_O_3_ on Cell Migration and Angiogenesis In Vitro

**Figure 3:**
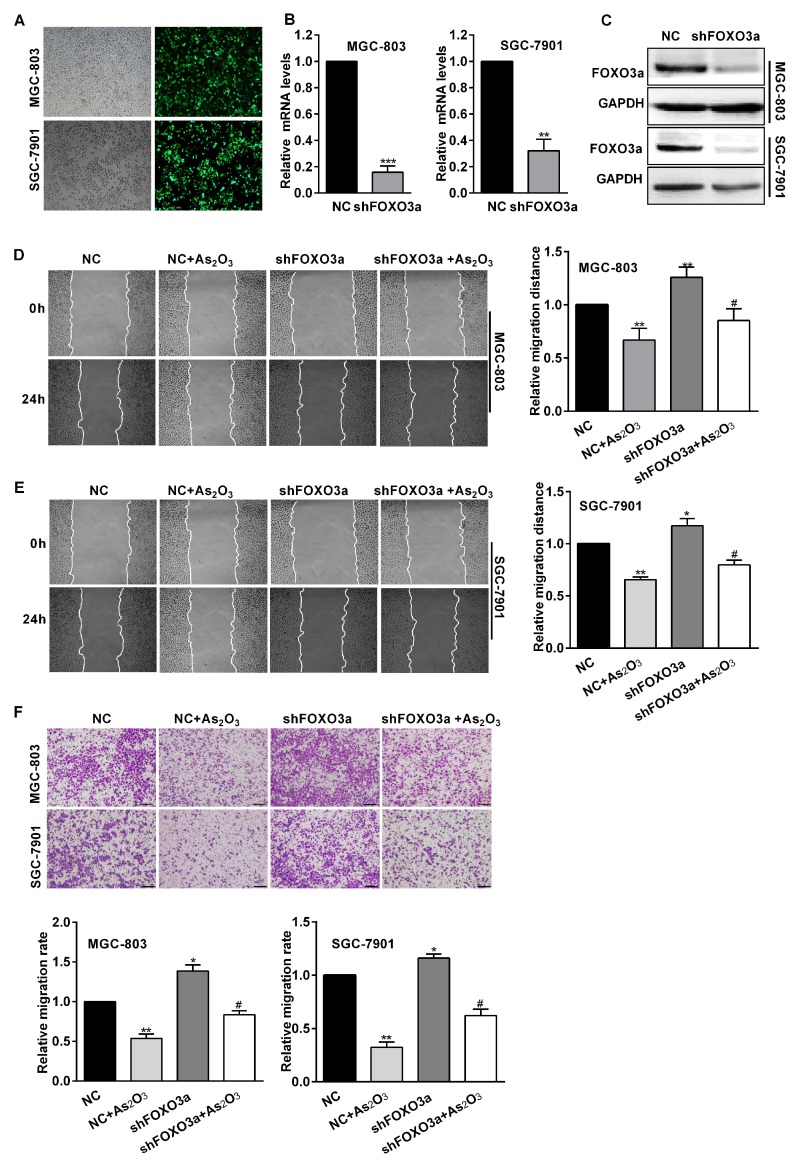
FOXO3a mediated the inhibitory effect of As_2_O_3_ on cell migration in vitro. (**A**) The cells transfected into the GFP-labeled virus were observed in white light and green fluorescence after transfection for 48 h (100×). (**B**) qRT-PCR analyses showed FOXO3a mRNA expression of MGC-803 and SGC-7901 cells infected with shFOXO3a or the negative control shRNA. (**C**) Western blotting analysis showed total FOXO3a protein expression of these transfected cells. (**D**,**E**) Wound healing assays showed the migration distances of transfected cells treated with As_2_O_3_ for 24 h (100×). The quantification of migration rates was analyzed respectively. (**F**) Transwell assays showed the migration of these cells treated with As_2_O_3_ for 24 h. The quantification of migration cells was performed. Scale bar, 100 µm. All data were obtained from three independent experiments. Compared with the NC group, * *p* < 0.05, ** *p* < 0.01, and *** *p* < 0.001; compared with NC + As_2_O_3_ group, # *p* < 0.05.

**Figure 4:**
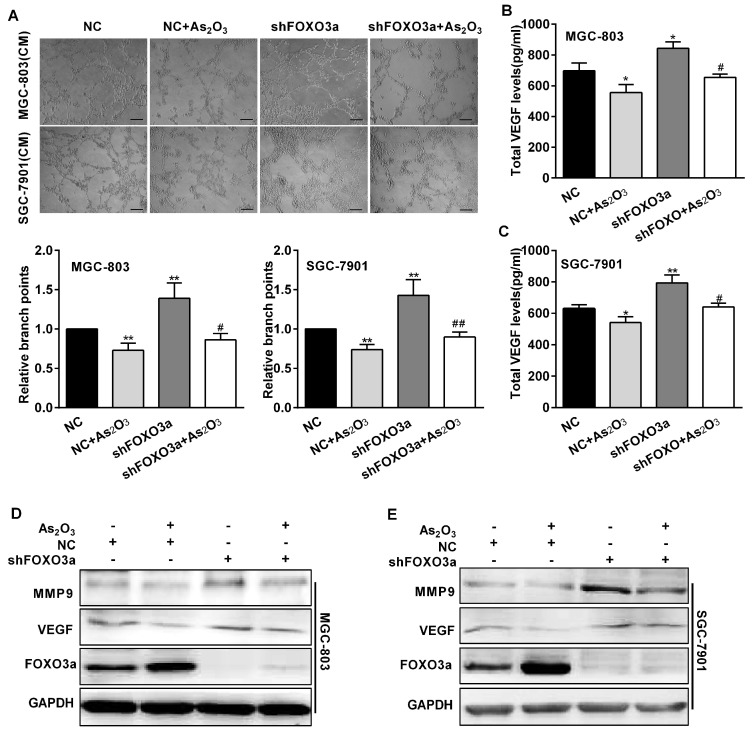
FOXO3a mediated the inhibitory effect of As_2_O_3_ on angiogenesis in vitro. (**A**) Tube formation assays detected formed tubes of HUVECs treated with CM from MGC-803 and SGC-7901 cells transfected with negative control shRNA or shFOXO3a. Calculating branch points per field was used to quantify the ability of tube formation. Scale bar, 100 µm. (**B**,**C**) ELISA analysis detected total VEGF protein secretion in these cells. All data were obtained from three independent experiments. (**D**,**E**) The expression of MMP9 and VEGF was detected by western blotting. Compared with the NC group, * *p* < 0.05, ** *p* < 0.01; compared with NC + As_2_O_3_ group, # *p* < 0.05, ## *p* < 0.01.

To assess the effect of FOXO3a in this process, MGC-803 and SGC-7901 cells were transfected with shFOXO3a or negative control shRNA (NC) before being treated with As_2_O_3_. The expression of GFP protein was found in more than 80% of transfected cells (Figure 3A). FOXO3a mRNA and protein expression levels obviously decreased in these cells transfected with shFOXO3a (Figure 3B,C). Wound healing assays showed knockdown of FOXO3a increased relative migration distances. Furthermore, compared with the NC and As_2_O_3_ group, the relative migration distances significantly increased in FOXO3a knockdown and As_2_O_3_ group (Figure 3D,E). Transwell assays also indicated that knockdown of FOXO3a led to a significant increase in cell mobility and participated in the inhibition of As_2_O_3_ (Figure 3F). Therefore, FOXO3a significantly reversed the inhibition of As_2_O_3_ on cell migration. Tube formation assays showed that knockdown of FOXO3a increased gastric cancer cells to induce tube formation of HUVECs, and also significantly repaired the inhibitory effect of As_2_O_3_ (Figure 4A). ELISA assays indicated knockdown of FOXO3a increased VEGF secretion levels and attenuated the inhibitory effect of As_2_O_3_ on VEGF secretion (Figure 4B,C). The results suggested FOXO3a mediated the inhibitory effect of As_2_O_3_ on angiogenesis in vitro. We continued to study the mechanism of FOXO3a in the inhibition of As_2_O_3_. Western blotting analysis showed knockdown of FOXO3a significantly increased MMP9 and VEGF expression and reversed the inhibition of As_2_O_3_ (Figure 4D,E). Accordingly, these results supported that FOXO3a participated in the inhibitory effect of As_2_O_3_ on migration and angiogenesis in vitro.

### 2.5. As_2_O_3_ Inhibited Gastric Tumor Growth and Angiogenesis In Vivo through Regulating FOXO3a

**Figure 5:**
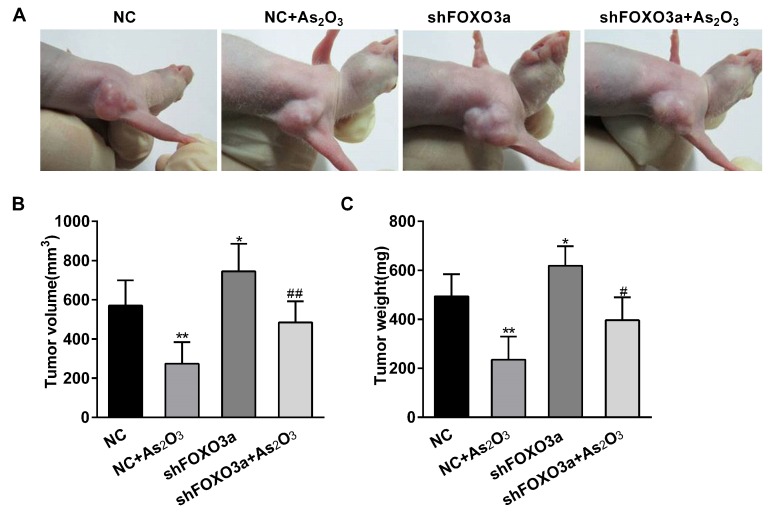
As_2_O_3_ inhibited gastric tumor growth through regulating FOXO3a. (**A**) The xenografted tumors were formed by subcutaneous injection with shFOXO3a or negative control shRNA MGC-803 cells in nude mice. The tumors were observed after As_2_O_3_ treatment. (**B**,**C**) The tumor volume and weight were respectively measured and statistically analyzed. Compared with the NC group, * *p* < 0.05, ** *p* < 0.01; compared with NC + As_2_O_3_ group, # *p* < 0.05, ## *p* < 0.01.

**Figure 6:**
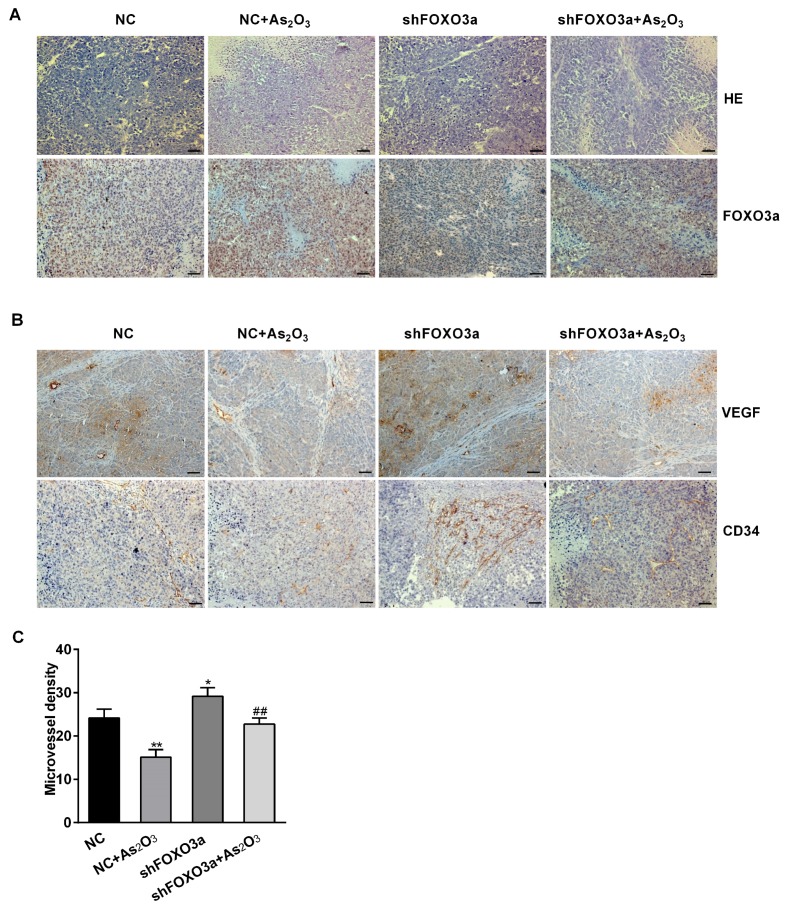
As_2_O_3_ inhibited tumor angiogenesis through regulating FOXO3a in vivo. (**A**) HE staining and immunohistochemical staining were used to analyze the structural features and FOXO3a expression in tumor tissues. (**B**) Immunohistochemical staining was used to analyze the expression of VEGF and CD34 in tumor tissues. Scale bar, 50 µm. (**C**) The microvessel density was evaluated by quantifying CD34 positive microvessels in tumor tissues. Compared with the NC group, * *p* < 0.05, ** *p* < 0.01; compared with NC + As_2_O_3_ group, ## *p* < 0.01.

To ascertain whether As_2_O_3_ played a role in the antitumor effect through regulating FOXO3a in vivo, we injected shFOXO3a- and negative control-shRNA-transfected MGC-803 cells into BALB/C-nu/nu nude mice. Seven days later, tumors formed significantly in the left fore armpits. Then, these mice were respectively treated with As_2_O_3_ (5 mg/kg/day) or the same volume of saline solution for 14 days. Tumor formation and gross morphology was observed in situ. The transplanted tumors were located under the skin, and the skin surface was intact without obvious rupture (Figure 5A). The tumors were weighed and measured after dissection. The excised tumor volumes and weights were analyzed statistically and represented graphically. Compared to those from the control group, the tumors derived from As_2_O_3_ group were smaller and lighter, while the tumors from FOXO3a knockdown group were larger and heavier. Compared with those from the negative control shRNA and As_2_O_3_ group, the tumor volumes and weights significantly increased in FOXO3a knockdown and As_2_O_3_ group (Figure 5B,C).Therefore, As_2_O_3_ inhibited gastric tumor growth by regulating FOXO3a in vivo. Hematoxylin-eosin (HE) staining showed that there was massive necrosis and less atypia in the tumor tissues treated with As_2_O_3_. However, there was less necrosis and more atypia and mitosis in the tumor tissues from FOXO3a knockdown group. These changes indicated that As_2_O_3_ had a certain therapeutic effect on tumor cells, while knockdown of FOXO3a significantly promoted the growth and invasion of tumor cells. To further understand the changes of FOXO3a and tumor angiogenesis in vivo, we used immunohistochemistry to detect FOXO3a and angiogenesis markers VEGF and CD34 levels in the tumor tissues. MVD was usually evaluated by the calculation of positively immunostained blood vessels with antibodies against CD34. The results showed the expression of FOXO3a significantly increased, while VEGF expression and MVD decreased in the tumor tissues treated with As_2_O_3_. Knockdown of FOXO3a obviously increased VEGF expression and MVD. Compared with those from the negative control shRNA and As_2_O_3_ group, knockdown of FOXO3a partially reversed the inhibitory effect of As_2_O_3_ (Figure 6B,C). Therefore, these results demonstrated that As_2_O_3_ inhibited gastric tumor growth and angiogenesis through enhancing FOXO3a expression in vivo. 

## 3. Discussion

Gastric cancer is one of the most common malignant tumors of digestive tract worldwide and exhibits a considerably high incidence and mortality with a dismal five-year survival rate [23]. Metastases are responsible for over 90% of deaths in patients with gastric cancer, but the molecular mechanisms that drive this process are not well understood. In the last decade, several anti-angiogenic drugs for cancer treatment have been approved and lately also introduced to gastric cancer treatment [24]. In the present study, our results revealed the previously unknown effect that As_2_O_3_ inhibited migration and angiogenesis of gastric cancer cells. 

As_2_O_3_, as an old drug, is mainly used in APL research and treatment [25]. In the past several decades, As_2_O_3_ began to be used in the study of solid tumors and was shown to have effective anticancer effects. As_2_O_3_ was shown to inhibit the proliferation and reduce the tumor sphere formation of lung cancer cells as well as reduce the expression of stem cell biomarker CD133 and transcription factors such as Sox2 and Oct4 [26]. As_2_O_3_ was also found to inhibit cell growth and motility via upregulation of let-7a in breast cancer cells [27]. As_2_O_3_ might act as an immune adjuvant in liver carcinoma treatment by increasing T lymphocytes and decreasing Treg infiltrated into tumors [28]. In addition, As_2_O_3_ induced human gastric cancer cells apoptosis by inhibiting the activity of anti-apoptosis-related factors and PI3K/AKT signaling pathway [29]. As_2_O_3_ has also been demonstrated to inhibit migration by regulating the expression of Cox-2/PGE2/MMP-2 in gastric cancer cells, and increased ROS played a critical role in this effect [30]. Furthermore, As_2_O_3_ attenuated STAT-3 activity and epithelial-mesenchymal transition (EMT) through induction of SHP-1 in gastric cancer cells [31]. As_2_O_3_ also reduced VEGF expression in a dose- and time-dependent manner to inhibit angiogenesis in gastric cancer cell xenografts [32]. However, the effect and mechanisms of As_2_O_3_ on migration and angiogenesis in gastric cancer have not been fully investigated. In this study, we found that As_2_O_3_ not only inhibited proliferation activity but also obviously inhibited the migration and angiogenesis of gastric cancer cells. These were consistent with the previous findings. Then, we explored the mechanism of As_2_O_3_ in the process.

The FOXO transcription factor family includes four members in humans: FOXO1, FOXO3, FOXO4, and FOXO6. FOXO transcription factors have been considered as tumor suppressors that limit cell proliferation and induce apoptosis in a limited number of human cancers [10,33,34]. FOXO3a has emerged as a versatile target for multiple disorders in cancers. In experimental studies, overexpression of FOXO3a inhibited the proliferation, tumorigenic potential, and invasiveness of cancer cells, while silencing FOXO3a resulted in marked attenuation in protection against tumorigenesis [35]. The increase of FOXO3a activities resulted in apoptosis and cell cycle arrest in breast cancer cells [36]. FOXO3a inhibited invasion and migration in prostate cancer cells, and it also inhibitedβ-catenin expression to suppress EMT of prostate cancer [37]. FOXO3a has been shown to attenuate in clear-cell renal cell carcinoma samples, and FOXO3a downregulation promoted renal cancer cell metastasis by upregulating SNAIL1 [38]. FOXO3a inhibited human gastric adenocarcinoma cell growth by promoting autophagy in an acidic microenvironment [39]. FOXO3a was a direct downstream target of AKT, and the activation of FOXO3a was a key function of AKT inhibition. FOXO3a in cytoplasm dephosphorylated and entered into nuclear localization, and hence promoted the transcription of its target genes [40]. In this study, we mostly searched whether FOXO3a mediated the process by which As_2_O_3_ inhibited gastric cancer migration and angiogenesis. 

We demonstrated that As_2_O_3_ suppressed the migration and angiogenesis of gastric cancer cells and exerted its anticancer activity partly via regulating FOXO3a. As_2_O_3_ obviously inhibited cell proliferation, migration, and tube formation in vitro. The effects were due to As_2_O_3_ decreasing VEGF and MMP9 expression. Meanwhile, As_2_O_3_ obviously increased total FOXO3a expression through attenuating p-AKT expression. Therefore, we speculated that As_2_O_3_ mainly played a role through the PI3K/AKT/FOXO3a pathway in this process. Knockdown of FOXO3a promoted cell migration and angiogenesis and partly reversed the inhibition of As_2_O_3_. In addition, we further found that As_2_O_3_ inhibited xenograft tumor growth in BALB/C-nu/nu nude mice, and knockdown of FOXO3a attenuated the anticancer effect of As_2_O_3_. The immunohistochemical staining showed that As_2_O_3_ effectively increased FOXO3a expression and decreased VEGF expression and MVD. However, knockdown of FOXO3a increased VEGF expression and MVD to promote tumor angiogenesis. Therefore, FOXO3a was significantly involved in the inhibitory effect of As_2_O_3._

## 4. Materials and Methods

### 4.1. Reagents and Antibodies

As_2_O_3_ was purchased from YIDA (Harbin, China). A stock solution of As_2_O_3_ was diluted into indicated concentrations when needed. The following primary antibodies were used: FOXO3a and CD34 were purchased from Abcam (Cambridge, MA, USA); AKT, p-AKT, P38, p-P38, ERK, and p-ERK were purchased from Cell Signaling Technology (Boston, MA, USA). VEGF and MMP9 were purchased from Proteintech (Wuhan, China). GAPDH and HRP-linked anti-rabbit secondary antibodies were purchased from Bioworld Technology (Louis Park, MN, USA).

### 4.2. Cell Cultures 

HUVECs and human gastric cancer cell lines MGC-803 and SGC-7901 were obtained from the Cell Biology Institute of the Chinese Academy of Sciences (Shanghai, China). Both of the cell types were cultured in DMEM medium (HyClone, Waltham, MA, USA) supplemented with 10% fetal bovine serum (FBS; Gibco, Carlsbad, CA, USA) in a humidified incubator at 37 °C with 5% CO_2_. All cells were routinely tested for the absence of mycoplasma contamination. 

### 4.3. Transient and Stable Transfection Studies

Lentivirus particles encoding shFOXO3a were purchased from GenePharma (Shanghai, China). SGC-7901and MGC-803 cells were cultured in 24-well plates at a density of 5 × 10^4^ cells per well. Cells were grown to 30–50% confluence and infected with lentivirus encoding shFOXO3a, green fluorescent protein (GFP), or control shRNA according to the manufacturer’s instructions. The cells were incubated for 24 h, and then the medium was replaced by 10% FBS DMEM medium. The intensity of GFP expression was observed under a fluorescence microscope after transfection for 48 h. The transfected cell medium was replaced by 10% FBS DMEM medium with puromycin after transfection for 72 h. The transfected cells were cultured with the medium containing puromycin for more than a week, and then they were used in subsequent experiments. 

### 4.4. Cell Counting Kit-8 (CCK8) Assay

To quantify the inhibitory effect of As_2_O_3_ on cellular viability, we used a commercial CCK8 assay kit (Dojindo Laboratories, Kumamoto, Japan) according to the manufacturer’s instructions. Cells were seeded (4 ×10^3^ cells) in 96-well plates. Then, the cells were treated with different concentrations of As_2_O_3_ for 24 h. For an additional 2-4 h, 10 μL of CCK8 was added to each well, and absorbance at 450 nm was measured. Inhibitory rates of cellular growth were calculated with the following formula: Inhibitory rate (%) = (1−OD value of experimental group/OD value of control group) × 100%. All the experiments were performed in triplicate.

### 4.5. Wound Healing Assay

The cells were equally seeded on a six-well plate chamber. When the fusion rate of cells reached 90%, a monolayer wound was made by scratching a 200 μL pipette tip along the bottom of the chamber. The medium was changed and the cells were washed twice with PBS. Along the vertical distances between both sides of the wound, at least four distinct, randomly selected areas were measured. Then, the cells were treated with or without As_2_O_3_ for 24 h, and the selected areas were measured again.

### 4.6. Cell Migration Assay

Gastric cancer cells were harvested, washed, and suspended in DMEM without FBS. Cells (2 × 10^4^/200 μL) were seeded into polycarbonate membrane inserts (8-μm pore size, Millipore, Billerica, MA, USA) in 24-transwell cell culture dishes. The lower chamber was filled with 600 μL of DMEM with 10% FBS. Cells were permitted to migrate for 24 h. The cells were washed, fixed, and then stained with 0.1% crystal violet. The stationary cells were removed from the upper surface of the membranes. The number of migrated cells to the lower surface was done by counting five regions of the filter under the microscope (OLYMPUS, Tokyo, Japan). The experiments were repeated three times.

### 4.7. Tube Formation Assay

A 96-well tissue culture plate was coated with growth-factor-reduced Matrigel (60 μL/well) to avoid bubbles on the ice. The plate was incubated at 37 °C for 30 min to solidify the Matrigel. HUVECs (2 × 10^4^ cells) were seeded in 96-well chamber slides and incubated in conditioned medium (CM) for 24 h. The numbers of endothelial tubes were examined under a light microscope (OLYMPUS, Tokyo, Japan) every 4 h by inspecting the overall branch points. The experiments were repeated three times.

### 4.8. Quantitative Reverse Transcription-PCR (qRT-PCR)

Total RNAs were extracted from cells using TRIzol (Generay, Shanghai, China). Quantified RNA samples were reverse-transcribed into complementary DNA (cDNA) according to HiScript^®^ II 1st Strand cDNA Synthesis Kit (Vazyme Biotech, Nanjing, China). AceQ qPCR SYBR Green Master Mix (Vazyme Biotech, Nanjing, China) was employed to quantify the target cDNA by using ABI StepOne™ Plus Real-Time PCR System (Applied Biosystems, Foster City, CA, USA) and analyzed using StepOne Software v2.1 (Applied Biosystemss, Foster City, CA, USA). The sequence of the primers for targeting molecules was designed as below: FOXO1, F: TCGTCATAATCTGTCCCTACACA, R: CGGCTTCGGCTCTTAGCAAA; FOXO3a, F: TCACGCACCAATTCTAACGC, R: CACGGCTTGCTTACTGAAGG; FOXO4, F: CCGGAGAAGCGACTGACAC, R: CGGATCGAGTTCTTCCATCCTG. The 2^−ΔΔ*C*T^ method was used to calculate gene expression levels. All samples were measured in triplicate relative to GAPDH values. 

### 4.9. Immunofluorescence Staining 

Cells were washed three times in PBS, fixed in 4% paraformaldehyde for 20 min, and permeabilized in 0.1% Triton X-100 for 5 min and blocked in PBS containing 1% BSA for 1 h at room temperature. The cells were incubated with primary antibody overnight at 4 °C. Then, the cells were incubated with FITC-conjugated secondary antibody for 1 h at room temperature. After being washed with PBS, the samples were mounted with DAPI for 5 min. The cells were observed under microscope (Olympus, Tokyo, Japan). 

### 4.10. ELISA Assay

SGC-7901 and MGC-803 cells were seeded in six-well plates. When the cell density was approximately 60–70%, the cells were treated with As_2_O_3_ for 24 h. Then, the cells were cultured in DMEM without fetal bovine serum. After 48 h of incubation, the culture supernatants were collected. The levels of VEGF protein secretion in the culture supernatants of tumor cells were quantified using the human VEGF ELISA Kit (R&D Systems, Minneapolis, MN, USA) according to the manufacturer’s protocol. They were analyzed in triplicate at 450 nm with a microplate reader. 

### 4.11. Western Blotting

Sample proteins of different treatment cells were harvested and quantified, and equal amounts of protein were loaded onto 10% SDS-PAGE gels, followed by electrophoresis and transferred to NC membranes. The membrane was blocked with 5% nonfat dry milk, then followed incubated with primary antibodies. Protein bands were detected by incubating with HRP-conjugated antibodies and visualized with ECL reagent (Millipore, Billerica, MA, USA). The intensity of bands was quantified using Image J software (National Institutes of Health, Bethesda, MD, USA). All data were representatives of at least three independent experiments.

### 4.12. Xenograft Models

Male BALB/C-nu/nu nude mice weighing 19–21 g (3–4 weeks old, Shanghai Institutes for Biological Sciences, Chinese Academy of Sciences, Shanghai, China) were used in this study. To evaluate in vivo tumorigenesis, MGC-803 cells (2 × 10^6^/200 μL) transfected with shFOXO3a or shRNA were injected subcutaneously into mice armpits subcutaneous of left forelimb. After seven days, the tumors reached approximately 100 mm^3^. Then, the mice in two groups were randomly divided into two groups that were injected with As_2_O_3_ (5 mg/kg/day) or the same volume of saline solution into the abdominal cavity every day. This concentration was based on our laboratory’s previous exploration of the concentration of As_2_O_3_ in mice. Eight mice per group were used for each set of experiments. After 14 days, four groups of mice were sacrificed and the tumor masses were collected. Care of experimental animals was in accordance with institutional animal care and use committee guidelines. All animal experiments were approved by the Experimental Animal Ethics Committee of Xuzhou Medical University (SYXK2015-0030, 14 March 2017).

### 4.13. Immunohistochemical Staining

Formalin-fixed, paraffin-embedded xenograft tissues were prepared. The paraffin sections were deparaffinized and rehydrated. Peroxidase blocking was done with 3% H_2_O_2_ in methanol for 15 min at 37 °C. The sections were processed for FOXO3a, VEGF, and CD34 antibodies overnight at 4 °C and then incubated with secondary antibodies for 1 h. Sections were visualized with DAB and counterstained with hematoxylin for microscopic examination. The staining intensity and the percentage of positive cells were multiplied to generate the immune reactivity score for each case. The MVD in tumor samples was measured according to CD34 staining and was calculated from the five most intensely vascularized areas with a magnification of ×200. The average value of the vessel count per field was defined as the final MVD value for each sample.

### 4.14. Statistical Analysis

GraphPad Prism version 6.0 software (GraphPad Software, Inc, La Jolla, CA, USA) was used for statistical analysis. Data were presented as mean ± standard deviation. The statistical significance was determined by a two-tailed Student’s *t*-test or one-way ANOVA. *p* < 0.05 was considered statistically significant.

## 5. Conclusions

In the present study, As_2_O_3_ inhibited migration and angiogenesis of gastric cancer cells via enhancing FOXO3a expression. The effect was mainly due to the regulation of PI3K/AKT/FOXO3a signaling pathway, which led to the decrease of MMP9 and VEGF expression. Together, these observations implied that As_2_O_3_ represented a potentially useful strategy for targeted cancer therapy. 

## Abbreviations

As_2_O_3_	arsenic trioxide
MVD	microvessel density
APL	acute promyelocytic leukemia
VEGF	vascular endothelial growth factor
HUVECs	human umbilical vein endothelial cells
CM	conditioned medium
